# Web-Based Health Services in Forensic Psychiatry: A Review of the Use of the Internet in the Treatment of Child Sexual Abusers and Child Sexual Exploitation Material Offenders

**DOI:** 10.3389/fpsyt.2018.00763

**Published:** 2019-02-04

**Authors:** Tamara S. N. Wild, Peter Fromberger, Kirsten Jordan, Isabel Müller, Jürgen L. Müller

**Affiliations:** ^1^Clinic for Psychiatry and Psychotherapy–Forensic Psychiatry, Human Medical Center Göttingen, Georg-August-University Göttingen, Göttingen, Germany; ^2^Prevention of Sexual Abuse (PsM), Asklepios Psychiatric Clinic Göttingen, Göttingen, Germany

**Keywords:** internet-based intervention, forensic psychiatry, child sexual abuse, child pornography, sex offender, pedophilia, web-based treatment, online therapy

## Abstract

In recent years, web-based health services for a variety of mental disorders have been developed and evaluated. Evidence suggests that guided internet-based therapy can be as effective as conventional face-to-face therapy. In forensic psychiatric practice, few web-based treatments have been implemented up to now. However, to our knowledge, there do not yet exist guided internet-based treatments for child sexual abusers and child sexual exploitation material offenders. This review aims at examining under what conditions patients are most likely to benefit from internet-based treatments. In addition, some computer-based health services in forensic psychiatry will be summarized and their potentials and weaknesses will be discussed. Subsequently, the review focuses on the implications for the development of online treatments for child sexual abusers as well as on a variety of ethical and legal issues that practitioners may encounter during the development, evaluation and delivery of online health services. The review will conclude with proposed quality standards for the development and implementation of web-based interventions for child sexual abusers and child sexual exploitation material offenders. By virtue of the low number of psychotherapists offering therapy to this clientele as well as individual barriers to seeking treatment such as fear of stigmatization, feelings of shame, long access routes, or limited mobility due to physical handicaps, the development of mental eHealth services in this sector could close an important healthcare gap. By increasing the density of supply, more child sexual abusers and child sexual exploitation material offenders would have the chance to engage in treatment and, ultimately, more incidents of sexual assault against minors could be prevented.

Child sexual abuse (CSA) is a worldwide spread public health problem defined as “[…] the involvement of a child in sexual activity that he or she does not fully comprehend, is unable to give informed consent to, or for which the child is not developmentally prepared and cannot give consent, or that violates the laws or social taboos of society. Child sexual abuse is evidenced by this activity between a child and an adult or another child who by age or development is in a relationship of responsibility, trust or power, the activity being intended to gratify or satisfy the needs of the other person.” (1). This broad definition states that CSA can occur on a continuum of power and control ranging from noncontact sexual assault (such as voyeurism, exhibitionism, forcing the child to listen to sexual acts) to contact offenses (such as genital fondling, forcible rape, making the child touch someone in a sexual way). CSA occurs to minors of all racial, social and age groups. Lifetime prevalence is estimated to range between 16.7–20% in women and 5.4–8% in men (2–4), but strongly depends on the definition used. As there are several limitations associated with self-report studies on delinquent behavior (5), it is difficult to estimate the population of sexual offenders against minors. Official arrest statistics, on the other hand, only include those offenders who are prosecuted for a crime. Due to reasons related to the sensitivity of the topic, also the prevalence of sexual interest in minors is difficult to investigate. Nevertheless, a Finnish twin study aimed at estimating the approximate prevalence of sexual interest in children and adolescents under the age of 16 in a sample of 3,909 men between 21 and 43 (6). In this cohort, 3% of participants reported a sexual interest in minors in the past 12 months, while 2.7% reported sexual fantasies with minors during masturbation within this time period. Additionally, 0.3% of participants indicated to have had sexual contact with a person under age 16. Notwithstanding, it has to be mentioned that results could be falsified due to self-selection bias, attrition bias, socially desirable responding, and fear of consequences (6).

Besides hands-on sexual offenses, the advent of new digital technologies and the growth of the internet have given rise to new forms of CSA: the sexual exploitation of children and adolescents online. As compared to the number of reported cases of CSA, a steep increase concerning the sexual victimization and exploitation of children in the online environment can be observed (7, 8). For instance, in 2016, 2,203 offenders were convicted for the distribution, acquisition, possession, and production of child or youth sexual abuse material according to §184b of the German penal code (9), whereas in 2002, it had only been 508 (10). Cooper (11) argues that the increasing numbers are a result of the easy accessibility of such materials online at affordable costs, while feeling secure due to the anonymity of the internet (the so-called Triple A Engine: accessibility, affordability, and anonymity). It is estimated that in 2009, 0.19–0.49% of search queries on *google* or *dogpile* were related to CSA material, meaning that one out of 200–500 queries was carried out in order to find such files (12). In 2013, search engines such as *google* or *bing* started to combat child sexual exploitation in the online environment, meaning that respective files were removed and warnings were generated whenever certain search queries were carried out. In contrast to other search engines that had not undertaken steps to block pedo-pornographic material, the amount of search queries related to CSA themes on mobile devices decreased by 67% (13). This does, however, not necessarily mean that these warnings led to a decrease in the consumption of abuse material. Instead, the drop most probably indicates a shift to other platforms such as chat rooms or peer-to-peer networks (13). Indeed, nowadays, peer-to-peer network sites are believed to be the major platforms for the distribution and acquisition of child sexual exploitation material (14). In a study investigating the distribution of child abuse files on the peer-to-peer file sharing network site Gnutella, Wolak et al. (15) found that in a one-year period, 120,418 pedo-pornographic files that had been identified as such by previous law enforcement investigations had been shared by 244,920 U.S. computers. In 2017, the Internet Watch Foundation (16) further recorded more than 3,000 domains containing images or videos of sexually exploited children, which amounts to an increase of 57% as compared to 2016. They additionally calculated that more than 50% of the URLs identified were hosted in the Netherlands and the United States.

Consequences for the victims are diverse and vary from a variety of mental health problems such as depression, suicide attempts, alcohol and nicotine dependence, social anxiety, conduct disorder, posttraumatic stress disorder, and bulimia nervosa to an increased risk for revictimization (4, 17, 18). While the effects of CSA on the psychological health of the victims are well-studied, data on the effects of the production and distribution of child sexual exploitation material is scarce (19). However, as behind every picture there is a child who had been abused (20), it can be hypothesized that the consequences are similar in nature. Yet, besides the consequences mentioned above, there is evidence that those victims may additionally suffer once they realize that their indecent images cannot be removed from the internet and are continuously being distributed and watched for sexual gratification by thousands of offenders (19, 21). By virtue of the high number of sexually abused children and adolescents, the detrimental effects on their psychological and physical health as well as on health care costs, and costs related to the imprisonment of the offenders, the prevention of (new) CSA is of high societal relevance.

## Sex Offender Treatment at Present

Therapeutic interventions for sex offenders, for example the *Sex Offender Treatment Programme* (22), to name one of the most commonly known, are usually based on the risk, need, and responsivity principles [RNR; (23)]. The RNR model asserts that therapeutic interventions are especially successful, (1) when treatment efforts are directed toward patients with a medium to high recidivism risk (risk principle), (2) when those risk factors are targeted that are the strongest predictors of recidivism (need principle) and (3) when the intervention adapts to the cognitive competencies and learning styles of the patient (responsivity principle). Cognitive behavioral therapy based on the RNR principles is the most commonly used and most effective treatment approach and is therefore considered the golden standard in the treatment of sex offenders (24, 25). The primary goal of treatment programs based on the RNR principles is the prevention of relapses (26). The focus of the RNR model lies on the identification of risk factors, risk situations, dysfunctional attitudes, and on an improvement of self-control strategies in such situations (27, 28). In line with previous meta-analyses (29), a recent meta-analysis comes to the conclusion that RNR-based treatment of child sex offenders is promising (24). The reported effect size was small, albeit statistically significant (*OR* = 1.41, *p* < 0.01), indicating that treatment reduced recidivism by 26.3 %. While untreated sex offenders recidivated at a rate of 13.7%, the recidivism rate of treated sex offenders was 10.1%. However, the authors remark that more studies using sophisticated research designs are needed to increase confidence in conclusions and to disentangle the working mechanisms underlying change.

With their Good Lives Model (GLM), Ward and Stewart (30) criticize the traditional RNR treatment programs. The central assumption underlying the GLM is that relapses can be prevented once the patients have the capabilities to fulfill their needs and achieve their goals in a prosocial way. The GLM can be understood as an add-on to traditional relapse prevention programs and emphasizes the importance of a positive therapeutic alliance and a non-confrontational therapeutic style. At present, the GLM is either integrated in treatment programs as an extra module or in the form of exercises throughout the treatment manual (31). Study results implicate that approach goals lead to higher engagement in therapy as well as therapist-rated motivation to live a life conforming to the law by the end of treatment as compared to avoidance goals (32). However, at this point in time, it is still unknown, if the implementation of the GLM also leads to lower recidivism rates (33). Accordingly, methodologically strong evaluation designs are needed to address the question of whether RNR-based treatment approaches augmented by GLM principles result in more favorable treatment outcomes (33).

## Who Seeks Treatment Finds an Open Door?

The provision of treatment for child sexual abusers (CSAs) and child sexual exploitation material offenders (CSEMOs) is allocated to correctional institutions, health care services, and specialized community programs. However, not all of those in need may find appropriate help. For instance, Stolpmann, Briken and Müller point out that for a number of reasons, effective intramural treatment turns out to be challenging (34): First of all, there is a lack of highly trained staff. As a consequence, treatment may not be administered according to best practice guidelines and standards. Second, sex offenders in general and CSAs and CSEMOs in particular may feel ashamed to disclose their offense in front of other inmates, which may, in turn, result in treatment failure. Third, psychotherapists working in correctional institutions are not bound to medical confidentiality. Consequently, patients may be reluctant to disclose information that may present them in a negative light (35).

In the community, probationers and parolees, who are mandated to treatment by the criminal justice system, might struggle finding a mental healthcare professional who accepts CSAs and CSEMOs. Indeed, a study with 86 German contractual psychotherapists revealed that 87.2% do not treat sexual offenders as a matter of principle (36). Only 2.6% indicated they would accept sexual abuse offenders, while 3.5% indicated that they would admit persons suffering from pedophilia. More than half of the practitioners justified the refusal with little knowledge of or experience in the treatment of sexual offenders, reservations regarding this patient group, and the fact that they also treat victims of sexual abuse (36). Other possible reasons are fear of relapses and reputational damage (37). Brand [as cited in (36)], on the other hand, argues that many psychotherapists avoid the necessary cooperation with legal authorities. These results are alarming as the literature suggests that the number of prior offenses, which often is higher in offenders who have been sentenced to imprisonment, correlates positively with recidivism risk (38) and that re-offense rates are highest in the first 2 years after prison discharge (39). Accordingly, access to extramural treatment for CSAs and CSEMOs has to be increased.

A possibility to decrease the discrepancy between demand and supply in the community would be specialized community aftercare supervision and treatment programs. However, while in the United States and Canada, community programs far outnumber residential treatment programs (40), availability in other countries is poor. For instance, outpatient treatment programs for CSAs and CSEMOs in Germany (e.g., the outpatient treatment center “*Prävention Sexuellen Missbrauchs*” [Prevention of Sexual Abuse; (41)], or the network “*Präventionsprojekt Dunkelfeld*” [Prevention Project Dark Field; (42)] were solely designed as prevention programs, meaning that they are not directed toward offenders with judicially imposed probation conditions and, in some cases, additionally also not toward offenders who are being prosecuted criminally. Although participation in these programs is, amongst others, related to a decrease in dynamic risk factor scores [e.g., the treatment center “Prevention of Sexual Abuse” reports a significant reduction of offense-supportive cognitions; *t*_(16)_ = 3.951, *p* = 0.001, *d* = 1.98; (43)], many CSAs and CSEMOs cannot participate in these programs as they do not fulfill the inclusion criteria mentioned above (44). However, as community-based treatment yields better outcomes than intramural treatment with regard to reducing recidivism risk (24, 45), an enhanced expansion of therapeutic services is necessary to achieve the best possible clinical outcome.

Like in the treatment of any other psychiatric illness, there exists a variety of other reasons contributing to the undersupply of individuals in need for help, ranging from long waiting-lists, and limited hours or mobility to shame-related issues and fear of stigmatization (46, 47). For instance, a survey revealed that some CSAs and CSEMOs would like to start therapy, but are scared of potential negative reactions on the part of the therapist (48). Another reason listed by the affected was that they doubt the psychotherapists' competence to treat individuals with a sexual interest in minors. In addition, 40% of those who had been rejected by mental healthcare practitioners in the past, felt discouraged due to something the practitioner had said to them (48).

## Closing the Healthcare Gap—The Development of Web-Based Treatments

In summary, a variety of reasons contribute to the undersupply of CSAs and CSEMOs. A possible solution to reach those who would like to start treatment but fail due to one of the reasons discussed in the section above, could be to develop an internet-based treatment that can be accessed at any-time from anywhere by an authorized person. There is a lot of variety concerning the set-up of web-based interventions (49, 50). While some interventions are psychoeducative in nature, others also include practical exercises and homework assignments that have to be completed offline and that will be reflected on during the next online session. Internet-based treatments also differ in terms of therapist guidance. In guided interventions, contact between health care professionals and clients can be synchronous, meaning that communication occurs in real-time, or asynchronous, meaning that counselor and client do not communicate directly, but time-delayed. Besides therapist-guided interventions, there also exist other internet-based programs that do not require guidance by a professional [e.g., psychoeducational self-help websites or peer-led forums; (50)]. Table 1 summarizes different types of web-based interventions as well as their advantages and disadvantages.

**Table 1:** Advantages and disadvantages of different types of web-based interventions adapted from (50–54).

**Intervention type**	**Advantages**	**Disadvantages**
Guided web-based interventions	• Therapist guidance • Personalized feedback • Time-saving • Cost-effective • No geographical barriers • No scheduling problems • Progress can be monitored automatically • Effectivity equivalent to f2f therapy	• Often mostly asynchronous contact
Videoconferencing	• Therapist guidance • Synchronous contact • Nonverbal communication possible • Personalized feedback • Progress can be monitored • No geographical barriers	• Time-consuming • Cost-intensive • Scheduling problems
Chat-based interventions	• Therapist guidance • Synchronous contact • Personalized feedback • Progress can be monitored • No geographical barriers	• Time-consuming • Cost-intensive • Scheduling problems
Secure e-mail communications	• Therapist guidance • Personalized feedback • Progress can be monitored • Scheduling problems • No geographical barriers	• Asynchronous contact • Provision of interactive trainings not possible
Psychoeducational websites	• Generally free to use • No scheduling problems • Low-threshold intervention • No geographical barriers	• No therapist guidance • No personalized feedback • Progress is not monitored by a professional
Forums	• Cost-efficient • Low-threshold intervention • Support from fellow sufferers • Foster a sense of personal empowerment and well-being • No geographical barriers	• Often no professional guidance • Danger of health misinformation • Other members may reinforce worrisome behavior • Progress is not monitored • Internet access necessary

Over the past years, dozens of randomized control studies aiming at investigating the effectivity of online treatments were conducted in the field of clinical psychology. In a meta-analysis, Hedman et al. (55) examined the effectivity of internet-based cognitive-behavioral therapies for a variety of mental disorders and found moderate to large effect sizes. For instance, the delivery of online therapy resulted in within-group effect sizes (pre- to post-treatment) between *d* = 0.38 and 2.27 in the treatment of depression, *d* = 0.62 and 2.92 in the treatment of panic disorder, *d* = 0.6 and 1.53 in the treatment of social phobia and *d* = 0.89 and 1.69 in the treatment of posttraumatic stress disorder. A possible explanation for the large differences in effect sizes for each mental disorder is, amongst others, the fact that the offered treatments differed with regard to frequency and intensity of the therapeutic contact. Indeed, several studies found that guided internet-based cognitive behavioral therapies are more effective than internet-based cognitive behavioral therapies without therapeutic guidance (56, 57) and that 10 min of support per patient and week are sufficient to achieve clinically significant improvements (58, 59).

In conventional therapies, a good therapeutic relationship is considered important for therapy outcomes (60). However, as the therapeutic contact between patient and therapist in web-based therapies has another character (e.g., no nonverbal communication) and the contact often only lasts 10 min per week as compared to 50 min in conventional treatment sessions (58, 59), a number of studies investigated therapeutic relationships and how they relate to therapy success in web-based therapies. Sucala et al. (61) discussed them in a literature review and concluded that the majority of patients perceive online therapies as being pleasant and personal. They additionally found that patients perceived the relationship between themselves and their therapist to grow in the course of the therapy sessions and that they did not miss face-to-face (f2f) contacts. Furthermore, they concluded that the quality of therapeutic relationships in web-based therapies does not differ from the quality of therapeutic relationships in conventional therapies. Besides, the authors could show that more than one modality of communication (for example chat and e-mail contact) leads to a more positive therapeutic relationship than only one communication modality and that also in web-based treatments, the quality of the therapeutic relationship correlates with treatment success. Andersson et al. (62) moreover compared conventional therapies and web-based therapies concerning their effectivity. However, for a variety of disorders such as social phobia, panic disorder, depression, dysmorphophobia, tinnitus, and sexual dysfunction in men, no differences were found. Summarized, guided internet-based therapies have been shown to be a cost-effective (55) and time-saving (58, 59) alternative to conventional therapies with comparable clinical efficacy (62).

With the beginning of the new millennium, the first research groups have started to integrate eHealth technologies in forensic psychiatry (51). With their online self-help program “Stop it now,” the Lucy Faithful Foundation (63) provides CSEMOs and individuals who are concerned they might consume such images or videos in the future with psychoeducative material to increase awareness of the problem. The website's content is, however, not intended to replace professional treatment. Additionally, to date, no evaluation studies have been published. Also in the field of offender treatment in general, studies on the implementation of computer-delivered interventions are scarce. Nevertheless, studies addressing dynamic risk factors that are also relevant for CSAs have been conducted with different offender groups. For instance, Levesque et al. (64) could show that perpetrators of domestic violence can benefit from brief computerized interventions. In their study, they compared domestic violence offenders participating in a court-mandated group treatment to domestic violence offenders who additionally received three computerized treatment sessions matched to their individual stages of change. They hypothesized that offenders in the brief intervention group would benefit more, as the intervention was tailored to the need of the participant (responsivity principle). Indeed, results indicate that this low-cost intervention had a favorable effect. At 5 months follow-up, perpetrators of domestic violence attending the extra sessions were more prone to seek help outside of their group and were better able to manage stress. Additionally, they were less likely to engage in physical abuse based on victim reports during the 12 month follow-up. They tended to be in a higher self-reported stage of change by the end of treatment but did not differ from the control group regarding treatment completion and further police involvement. In summary, this promising outcome highlights the effectivity of time-saving and cost-effective interventions as an adjunct to usual care for offending populations. Another benefit of the program tested in a pilot study (65) is that the online program was found to be informative, helpful and easily usable. Additionally, the majority of participants believed that the online sessions could help them change problematic behaviors and indicated that they would return to the program in the future (65). Future research will nevertheless have to investigate, if therapist-guidance would have led to increased intervention effects. Considering that previous research found guided treatments to be superior to unguided treatments (56, 57), this seems to be a plausible assumption.

Evidence suggests that cognitive behavior therapy based on the RNR principles is the most effective treatment approach for CSAs and CSEMOs (24, 25, 29). Yet, to our knowledge, no internet-delivered cognitive behavioral therapy-based intervention study targeting sexual behavior of offenders has been conducted so far. Nevertheless, a research group has incorporated cognitive behavior therapy together with the community reinforcement approach in a computerized intervention for inmates suffering from drug addiction disorders (66). The aim of this study was to assess the effect of this treatment approach on treatment utilization, skills acquisition and treatment satisfaction as compared to standard care. The experimental condition consisted of 48 online-modules that were delivered 2 h a week over a period of 12 weeks and were comprised of topics such as risk reduction, coping skills, and emotion regulation. Standard care, on the other hand, consisted of substance abuse treatment provided by a certified addiction counselor and was comparable concerning length and dosage of the intervention. In total, 494 male and female inmates, who had been randomly assigned to either of the conditions, completed the study. Results indicate that the number of attended sessions did not differ significantly between the two groups. On average, half of the prescribed sessions were attended. Also concerning skills acquisition, no differences between the two groups were found. In both groups, coping skills improved significantly from baseline to 3 and 6 months post-prison release. Inmates in the computer-based intervention group, however, perceived their treatment as more interesting and more satisfying and felt that they had received more new information as compared to the control group. These results show, that also in in-patient settings, computer-based interventions can be as effective as conventional standard care and are even considered to be more satisfactory. However, as the study did not include any long-term outcomes, future research should investigate the effects of computerized interventions on recidivism and compare the findings to the effects of conventional treatment.

The only internet-delivered cognitive behavioral therapy-based intervention study targeting sexual behavior that we know of was conducted with a non-forensic sample, namely individuals suffering from porn addiction (67). In this study, 211 participants completed 10 online modules including psychoeducative text material, interactive exercises, and videos aiming at reshaping thought processes in order to alter the addictive behavior. Retrospective self-report data indicated that the online treatment was found to be at least as helpful as any other form of treatment that the participants had engaged in in the past. After 26 weeks of treatment, both the usage of pornography and excessive masturbation had decreased while improvements on a variety of measures such as sexual preoccupation and perceived control were observed [(67), Hardy, Ruchty, Hull and Hyde as cited in (68, 69)]. A first limitation of this study is that no randomized controlled trial was carried out to compare the effects of the online program to conventional treatment or no treatment. Second, the study purely relied on retrospective self-report measures, and therefore, the results may have been affected by problems with accurate recall or desirable responding. Nevertheless, the findings are promising and suggest that an adapted treatment approach may also be effective in the treatment of CSAs and CSEMOs.

In summary, it can be concluded that online health services are effective ways of treating a variety of mental disorders. Also in forensic psychiatry, computer-delivered interventions have been shown to lead to improvements in different offender populations. In addition, empirically supported and promising risk factors related to recidivism in sex offenders including but not limited to emotion regulation, sexual preoccupation, and dysfunctional coping have been successfully addressed in computer-based treatments for other offender groups and non-forensic individuals. By virtue of these promising results, it is plausible to assume that also CSAs and CSEMOs could benefit from such treatments. In the following, advantages and limitations related to online interventions for this clientele will be discussed.

## Advantages of web-based Therapies for Child Sexual Offenders and Ethical and Legal Considerations

### Increased Access to Mental Health Care

Both in general psychiatry and in forensic mental health, technological interventions have been shown to be effective in the treatment of a range of psychological disorders (51, 55, 67). Below, advantages of web-based interventions for CSAs and CSEMOs as well as some ethical and legal aspects will be discussed and possible solutions will be offered [for an overview, see Table 2; for reviews on advantages and limitations of internet-delivered therapies in general, see (46, 51–54)]. A first advantage reported by Kip et al. (51) was that eHealth technologies, including web-based interventions, can increase access to mental health services in forensic and legal settings. Indeed, this is both the case for outpatients and incarcerated offenders. For instance, web-based interventions could eliminate financial and staff-related barriers in correctional institutions (labor shortage, high patient caseloads, insufficient training) and, as a consequence, increase the supply of health services. One could argue that incarcerated CSAs and CSEMOs should not be allowed to engage in internet-delivered interventions as they might misuse the internet for criminal purposes. However, this problem can be easily solved by restricting access to websites other than the intervention site, as has been done by Chaple et al. (66). Also in outpatient child sexual offenders, the delivery of online interventions would increase access to health care for a variety of reasons. For instance, via online interventions, geographical barriers could be overcome as individuals living in remote areas with a low therapeutic density, as well as individuals with limited mobility or limited access to means of transport would have the possibility to start treatment.

**Table 2:** Advantages of and challenges in guided web-based interventions for child sexual abusers and child sexual exploitation material offenders.

	**Advantages**	**Challenges**
For patients	Feelings of anonymity	Computer, tablet or smartphone access is necessary
	Increased access to mental health care for individuals • From areas with a low therapeutic density • With limited physical mobility • With limited access to means of transport • Who are being prosecuted criminally • With judicially imposed probation conditions	Internet connection problems Possible solution: elaborate an emergency kit containing important skills together with the patient in the beginning of the intervention; offer alternative means of support (e.g., by telephone)
	No scheduling problems	Little or no knowledge on computer usage Possible solution: provide users with tutorials
	Less fear of stigmatization	
	Computerized treatments are often perceived as satisfactory	
For therapists	Lower therapist workload	No direct reaction possible Possible solution: in the case of exercises, automatic corrections can be generated by default; synchronous chat application can be placed on the intervention site
	Therapists can inform themselves or consult colleagues before answering	The intervention has to suit both highly educated and poorly educated individuals Possible solution: barrier-free website design to ensure that the website is accessible to all users
	Time-saving • Less time investment needed • Information given by the patient is stored and results arecalculated by default • Treatment progress can be monitored more easily more timefor additional patients	Lack of evaluated risk assessment tools Possible solution: development of an online risk assessment tool or f2f risk assessment
	Increased treatment fidelity	High demands on data security Possible solution: encryption, hardware and software solutions, pseudonymisation, etc.

Besides the barriers mentioned above, feelings of stigmatization in individuals suffering from mental disorders in general, and child sexual offenders in particular (47, 70), contribute to the undersupply of this patient group. Due to feelings of anonymity, the online environment might offer “a safe place” for those who would be too scared to disclose to a therapist in f2f settings or in front of other inmates during group therapies in correctional institutions. In addition, also those who wanted to start with a conventional therapy but were rejected by mental health professionals, for example because they are criminally prosecuted or because they have to fulfill judicially imposed probation conditions, could finally engage in therapy. In summary, with interventions delivered over the internet, offenders who would otherwise have remained untreated could get increased access to mental health care.

### Website Accessibility

Universal design is defined as “the design of products and environments to be usable by all people, to the greatest extent possible, without adaptation or specialized design” (71)^1^ This broad definition refers to a wide range of design disciplines and also includes website accessibility. According to the principles of universal design, all CSAs, and CSEMOs, regardless of the type of electronic device they use or their cognitive abilities or potential physical disabilities, should be able to use the intervention website. To increase the number of potential users, we propose to design and build the intervention site using responsive web design techniques, so that it can be used from different devices, including desktop computers, laptop computers, tablets, and smartphones. Of course, access to the internet is a precondition for any online treatment. However, as ~76% of U.S. Americans, 90% of Swedes and Germans, and 95% of the British have had access to the internet in 2016 (72), it can be assumed that at least in industrialized countries, the majority of those in need could be reached. Nevertheless, tutorials on how to use the intervention site should be provided to ensure that also those with little computer experience can participate. The website itself should be designed barrier-free: Navigating the website should be intuitive and self-explanatory, non-essential physical effort should be minimized and the design should accommodate a wide range of individual preferences and abilities (71). Differences in cognitive abilities further impose some requirements on the set-up of online therapies. In a review of the current understanding of web design guidelines, Friedman and Bryen (73) summarize recommendations for websites that also address individuals with cognitive disabilities, some of which may also be useful in the development of online interventions for CSAs and CSEMOs.

### Risk Management in Online Interventions

Risk management following the RNR principles is a crucial component in the treatment of sex offenders as it allows mental health care professionals to assess changes in individual recidivism risk over time. According to the risk and need principles of the RNR model, the patients' individual recidivism risk as well as dynamic risk factors must be assessed before and during the treatment to determine if the intervention is adequate for the client with regard to his offense, to identify treatment targets, and to adjust levels of control accordingly. However, with regard to online interventions, one major disadvantage is the lack of evaluated online risk assessment tools. Accordingly, research groups evaluating internet-based services for this clientele and professionals providing CSAs and CSEMOs with online interventions will either have to develop such a tool first or they will have to rely on conventional methods of risk assessment such as the Stable-2007 and the Acute-2007 (74). However, the last option would require f2f contacts between therapist and patients. We therefore propose that online risk assessment tools should be developed and evaluated before any intervention for sex offenders is offered online.

Throughout the therapy, patients may have recurrent deviant sexual fantasies concerning children or adolescents. Therefore, in the beginning of the online intervention, an emergency kit containing important skills to overcome such situations as well as a list with emergency numbers should be elaborated together with the patient, so that in case of an emergency, the patient knows what to do or who he can turn to (a friend, family member, a psychiatric clinic, etc.). This procedure has been shown to be effective in online interventions for patients with other psychiatric disorders who needed support as fast as possible [e.g., depression and suicidal behavior; (75, 76)]. Furthermore, we propose to include some kind of measure assessing sexual urges in the beginning and in the end of each therapy session. In case patients indicate that they have distressing fantasies and are afraid they might relapse, therapists should receive an automatic message that they should contact their client. Contact could be established via a chat function on the intervention website or by telephone. Like this, patients would have the chance to refresh their knowledge on risk management strategies together with their therapist and to regain confidence in their ability to resist the temptation. A clear advantage of online emergency risk management sessions is that no f2f sessions are necessary, meaning that problems concerning distance or mobility can be bypassed. Also scheduling problems play a less important role, as patients would not need to come to a psychotherapeutic practice, but could instead attend the appointment from home, work, or anywhere else, using a (laptop) computer, tablet, or smartphone. Of course, the methods mentioned in this paragraph cannot replace traditional and evaluated risk assessment tools and risk management plans. Nevertheless, they may be a possibility to provide patients with tools and skills that they might find useful in controlling sexual urges and, ultimately, to help them overcome risk situations.

### Handling of Comorbid Disorders

Mental health care professionals have to assess if interventions are suitable with regard to potential comorbid disorders. However, up to now, there do not yet exist any valid online psychological assessments for CSAs and CSEMOs. As a consequence, at this point in time, a professional diagnostic procedure like in the case of f2f treatment is also essential for online interventions. Thus, each client should be seen f2f before an online intervention is offered to him. This does, however, not mean that patients have to cover long distances to see the professional who also guides them through the intervention before the start of the online program. Instead, the psychological assessment could be administered by any psychotherapist in the patients' neighborhood. As they would not be involved in the treatment itself, also those who have refused to work with sex offenders in the past because of little knowledge of or experience in the treatment of this clientele may agree to accept CSAs and CSEMOs.

Due to the nature of online interventions, comorbid disorders developing in the course of treatment might not always be noticed by online therapists (54). We therefore suggest the use of online questionnaires assessing the presence of some of the most common comorbid disorders on a regular basis. Fromberger et al. (77) summarized that a rather large proportion of pedophile sex offenders suffer from affective disorders, anxiety disorders, addiction, personality disorders, and other disorders of sexual preference. These comorbid disorders are not only prevalent in individuals with a sexual interest in children, but also in sex offenders in general (78). Additionally, some offenders fulfill the diagnostic criteria for sexual dysfunction, psychosis, and attention-deficit/hyperactivity disorder (78). As major depressive episodes or severe addictions may result in high levels of psychological distress, self-endangerment and endangerment of others, and may therefore interfere with the online intervention, it is important to regularly assess the extent of these disorders during the course of the intervention. Accordingly, questionnaires on these mental illnesses should be administered subsequent to or following some of the online sessions. By administering them online, time can be saved since results can be calculated and compared with representative samples in real-time. In the case of clinically significant scores, the therapist should contact the patient to determine if the treatment of the comorbid disorder should have priority. Treatment of interfering comorbid disorders could either be provided by psychotherapists in f2f settings or by tailored internet-based treatments (79). That is, modules addressing the individual disorders, for instance depression, could be unlocked and the patient could be asked to work on these before proceeding with the actual intervention.

### Therapist Guidance

Web-based interventions have been shown to be a time-saving alternative to conventional therapies. Even though evidence suggests that the most effective online treatments require guidance by a psychotherapist (56, 57), the time spent on each patient is less than in conventional therapy (58, 59). As a consequence, therapists have more time that they can devote to the treatment of additional patients. Another factor contributing to the saving of time is that in online interventions, information given by the client is stored automatically and outcome scores to monitor treatment progress can be calculated by default (54). The immediate calculation of problematic scores would give the therapist the possibility to intervene as soon as possible. For instance, he or she could seek contact via a chat function on the website, via phone or in the case of incarcerated individuals also in a f2f therapy session. As more than one modality of communication has been related to a better therapeutic relationship and the therapeutic relationship may positively relate to therapy success (61), mental health care specialists should provide at least two communication modalities.

### Enhancement of User Motivation

In their meta-analysis, Mann et al. (80) reported that motivation does not predict recidivism in sex offenders. Nevertheless, enhancing patient motivation has several benefits including decreased attrition rates (81). As treatment participation correlates negatively with recidivism (24), also in web-based interventions, motivation should be targeted in order to enhance treatment adherence. In the traditional treatment of psychiatric disorders, mental healthcare professionals often address patient motivation by methods such as motivational interviewing. This approach could also be used in web-based interventions for CSAs and CSEMOs. For instance, in the beginning of the intervention, a module could be provided that addresses motivational factors. Based on the findings by Levesque et al. (64), the content of the module should be matched to the patient's individual stage of change. However, next to traditional methods of motivational enhancement, internet-based therapies additionally allow for more advanced motivational approaches. That is, like in the case of serious games (computer-based learning environments that use entertainment to convey learning and skills) elaborate reinforcement and reward schedules can be used to maximize motivation. Indeed, research suggests that computer games result in higher cognitive gains and more favorable attitudes toward learning as compared to traditional teaching methods (82). We therefore propose the utilization of game-based virtual incentives throughout the online intervention.

### Technological Considerations, Data Security, and Data Protection

Kip et al. (51) further conclude that in forensic mental health, eHealth technologies including interventions delivered over the internet are judged as being pleasurable by the majority of patients. While some were not enthusiastic about using technology, most held positive opinions regarding this new form of treatment. However, common technological issues such as disturbed internet connections or other computer problems can occur (83, 84) and pose challenges for online counseling providers. Riemer-Reiss [as cited in (46)] therefore suggests that in cases like these, alternative means of therapy such as phone counseling or f2f contacts should be offered. An important topic related to the use of technology that was also evident in some of the studies reviewed by Kip et al. (51) were concerns about privacy. Data protection has received and continues to receive elaborate attention, and new laws have been passed recently. For instance, the EU General Data Protection Regulation [GDPR; (85)] entering into force as from May 2018 requires mental healthcare specialists offering internet-based therapies to “take into account the state of the art, the costs of implementation and the nature, scope, context and purposes of processing as well as the risk of varying likelihood and severity for the rights and freedoms of natural persons, … [and to] implement appropriate technical and organizational measures to ensure a level of security appropriate to the risk” (Article 32 GDPR). By means of these preoccupations, the patients' privacy shall be protected. This is especially important for CSAs and CSEMOs, as the effects of data leaking could be detrimental, ranging from social exclusion, and violent threats to loss of housing or employment and suicidal thoughts and behavior (86–88).

Common standards concerning consent in mental health care can be summarized as follows (52): Psychotherapists have to ensure that consent forms are valid and that patients fully understand the information given to them, including potential risks and benefits of the offered service. Additionally, they have to ascertain that patients are not forced or pressured into giving consent (52). In internet-based therapies without f2f diagnostics, this is a challenging endeavor as therapists have never met their patients personally. Accordingly, deciding on whether or not a person is under the influence of alcohol or other drugs, suffers from transient psychotic episodes or does simply not have the literacy skills or cognitive abilities to understand the consent form is difficult (46, 52). However, this is only of concern for professionals deciding to offer online interventions to individuals who are unknown to them. As we have mentioned before, we believe that it is essential to see clients before an online intervention is offered to them, as it has to be determined if treatment is adequate with regard to the offense and potential comorbid psychiatric disorders.

## Proposed Quality Standards for Online Interventions Targeting CSA

**Figure 1:**
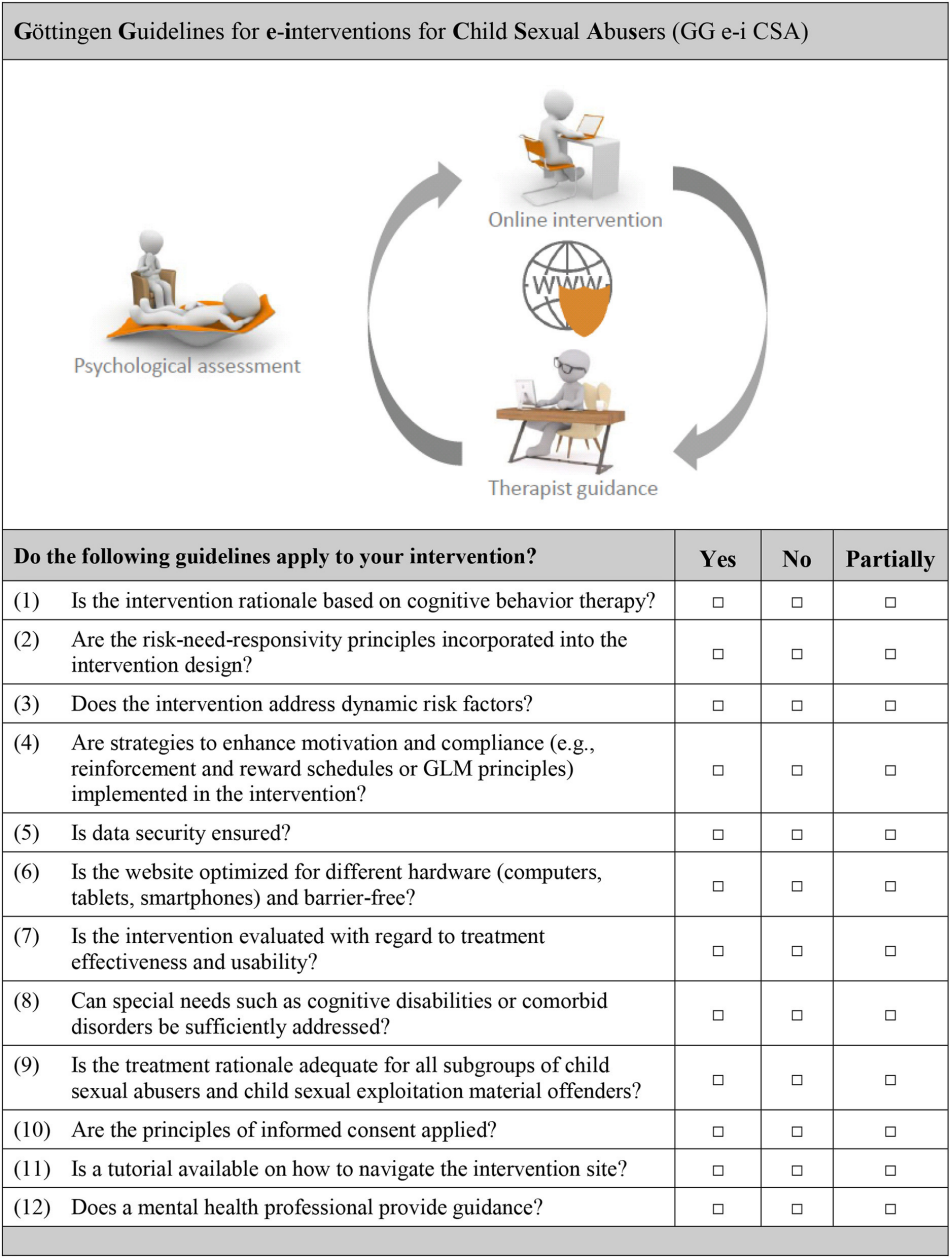
Quality criteria checklist for the development and implementation of web-based interventions for child sexual abusers and child sexual exploitation material offenders.

By virtue of the possibilities and pitfalls mentioned above, we propose the following work-flow for online interventions for CSAs and CSEMOs: (1) Cognitive behavioral therapy should be the treatment of choice (24). (2) Based on current knowledge about effective f2f treatments for sex offenders, a consequent and continuous risk-management following the RNR principles is essential. We therefore propose that risk management based on the RNR model should also be of major consideration in the development of web-based interventions for this clientele (24). (3) The intervention should address dynamic risk factors that have been shown to relate to recidivism risk (80). As regards content, established treatment methods of f2f treatment programs [e.g., (22, 42, 89, 90)] addressing dynamic risk factors could be adapted for use in the online environment. Additionally, guidelines for therapeutic work with internet sex offenders (91) should be implemented. (4) Even though one's motivational state has not been identified as a risk factor for recidivism (80), lack of patient motivation predicts dropout rates in the treatment of sex offenders (81). In order to reduce attrition rates in online interventions, motivation shall be increased by using traditional motivational interviewing techniques. However, online interventions additionally allow for more advanced motivational approaches. For instance, like in the case of computer games, elaborate reinforcement and reward schedules can be used to maximize motivation. To further increase user compliance, GLM principles should be implemented throughout the intervention (32). (5) Following state-of-the-art protocols, data security, and data protection should be ensured (85): All communication should be encrypted and databases should be secured not only by software but also by hardware solutions. Personal data should, at no point, be digitally stored or transmitted. Moreover, other precautionary actions such as pseudonymisation or data access control shall be used to reduce risk and scale of breaches. (6) To increase accessibility, state-of-the-art responsive web design techniques shall be used to design and build the website. Herewith, users have the possibility to access the intervention site using different devices such as desktop computers, tablets, and smartphones. (7) Every mental health treatment program, including internet-based interventions for CSAs and CSEMOs should be thoroughly evaluated before it is made available to the public. That is, it has to be ensured that the intervention is safe with regard to data security and does not have any unwanted side-effects such as an increased urge to (re)offend or suicidal thoughts or behaviors. To enhance user confidence in online interventions, this is crucial, especially when considering that nowadays, a large number of mobile apps are flooding the market. We propose that first evaluation studies should be conducted both with incarcerated CSAs and with outpatients who, until an online risk-assessment tool is developed, agree to attend regular f2f risk assessment sessions. Possible side-effects should further be assessed continuously. (8) As it is true for f2f-treatments, online interventions may not be adequate for all CSAs and CSEMOs. For instance, individuals with low cognitive abilities and users suffering from comorbid disorders that are not manageable by the program may not be able to use the program in the intended way, which may in turn result in treatment failure. We therefore propose the development of additional online modules that can be unlocked for users in need or the development of adapted online interventions for specific user groups. In either case, common web design guidelines by Friedman and Bryen (73) should be considered. In order to provide the best patient-centered care possible, f2f-meetings are, in our opinion, at the moment the only possibility to decide on whether the online intervention or an alternative form of treatment is indicated. (9) As the literature suggests that not more than 50% of sexual offenses against minors are committed by individuals who fulfill the diagnostic criteria for pedophilia (92), participation in the intervention should be offered to those who have committed a sexual offense against minors or who fear that they might commit such an offense in the future, irrespective of whether a sexual interest in minors is present or not. (10) In addition to the above mentioned data security and data protection protocols, informed consent following traditional principles of psychotherapy is also essential in online interventions. Before the start of the intervention, the user must be informed about the content of the intervention, risks and benefits of participating in the program, the tasks he has to do, the kind of data that is saved and what will be done with this data. (11) Based on the principles of universal design (71), a tutorial on how to navigate the intervention site should be provided to users, especially to those with little computer knowledge. (12) Since guided web-based interventions have been shown to be more effective than self-help programs (56, 57), the online intervention should include guidance by a mental healthcare specialist. That is, professional coaches guide each user individually through the online intervention and provide help promptly or in real-time. Guidance could include help with comprehension, feedback on exercises and assignments and stimulation of critical reflection. Based on the RNR-principles, the therapist should spend more time on high risk offenders, especially address risk factors related to recidivism and should be responsive to the patients' individual abilities, competencies and strengths. Additionally, more than one modality of communication should be used. Figure 1 summarizes the ***G***ö*ttingen* ***G**uidelines for* ***e**-**i**nterventions for* ***C**hild* ***S**exual* ***A**bu**s**ers* (GG e-i CSA) in a quality criteria checklist.

## Conclusion

In summary, there are a substantial number of ethical and legal issues that have to be considered during the development, evaluation, and implementation of online health services for CSAs and CSEMOs. Nevertheless, web-based treatments in forensic mental health have a number of advantages including increased access to health care, cost-effectiveness, time-savings, positive opinions regarding the use of technology and increased fidelity. Accordingly, we argue in favor of the development of an internet-based cognitive behavioral program for child sexual offenders using the presented quality standards. Sophisticated evaluation studies will have to investigate if interventions delivered over the internet have the potential to reduce recidivism as a stand-alone treatment or if they have additional beneficial effects on treatment outcomes when used as an adjunct to conventional f2f treatments.

## Author Contributions

PF and JM conceived the topic of the review. TW and PF conducted literature searches and TW wrote the first draft of the manuscript. PF and JM supervised the writing process and, together with KJ and IM, critically revised the manuscript and approved the final version.

### Conflict of Interest Statement

The authors declare that the research was conducted in the absence of any commercial or financial relationships that could be construed as a potential conflict of interest.

## Footnotes

^1^https://projects.ncsu.edu/design/cud/pubs_p/docs/poster.pdf
